# Role of 4-aminobutyrate aminotransferase (ABAT) and the lncRNA co-expression network in the development of myelodysplastic syndrome

**DOI:** 10.3892/or.2022.8348

**Published:** 2022-06-14

**Authors:** Yanzhen Chen, Guangjie Zhao, Nianyi Li, Zhongguang Luo, Xiaoqin Wang, Jingwen Gu

Oncol Rep 42: 509–520, 2019; DOI: 10.3892/or.2019.7175

Following the publication of the above paper, the authors have realized that the cell apoptosis and cell proliferation assays in Fig. 8 were poorly presented, which made the interpretation of the data difficult. Furthermore, a change was also required to the text concerning the description of Fig. 8: The sentence starting on p. 517, left-hand column, line 7 (‘The fraction of apoptotic cells was 22.41±2.596 in the lncENST00000444102-overexpressing SKM-1 cells, and 8.650±0.889 in the negative control; the fraction of apoptotic cells was 20.58±2.190 in the lncENST00000444102-overexpressing THP-1 cells and 8.192±0.997 in the negative control group (P<0.001, Fig. 8B)’ should be replaced with the following text: ‘Flow cytometry showed that the fraction of apoptotic cells increased in the lncENST00000444102-overexpressing SKM-1 and THP-1 cells, as determined by Annexin V-APC/7-AAD staining at 48 h (P<0.05; Fig. 8B)’.

A revised version of Fig. 8, presenting the results of the flow cytometric analysis more clearly, is shown on the next page. Note that the revisions made to this figure have not had a major impact on the reported results, and do not affect the overall conclusions reported in the study. All the authors agree to the publication of this corrigendum. The authors are grateful to the Editor of *Oncology Reports* for allowing them the opportunity to publish this Corrigendum; furthermore, they apologize for any inconvenience caused to the readership of the Journal.

## Figures and Tables

**Figure 8.**
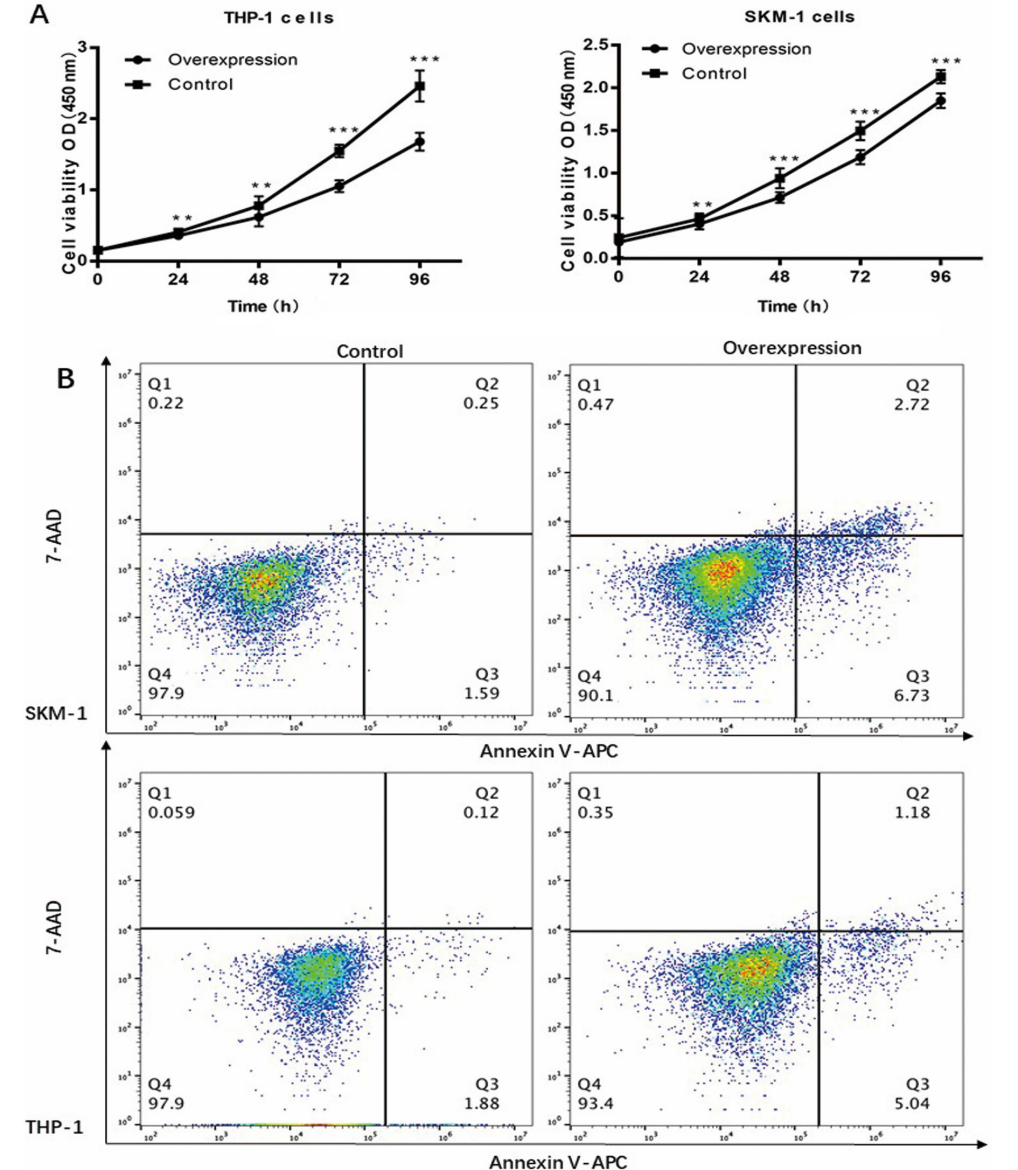
Reduction in MDS cell viability and induction of apoptosis after lncENST00000444102 overexpression. (A) Stable lncENST00000444102-overexpressing THP-1 and SKM-1 cells were subjected to cell viability CCK-8 assay. Data are presented as the means ± standard deviation. (B) Stable lncENST00000444102-overexpressing SKM-1 and THP-1 cells were subjected to flow cytometric apoptosis assay. Apoptosis of SKM-1 and THP-1 cells with lncENST00000444102 overexpression, as determined by Annexin V-APC/7-AAD staining at 48 h. MDS, myelodysplastic syndrome; 7-AAD, 7-amino-actinomycin D; APC, allophycocyanin; sh/shRNA, short hairpin RNA.

